# (2*E*)-1-[2,3-Dichloro-6-methyl-5-(trifluoro­meth­yl)phen­yl]-2-(1-phenyl­ethyl­idene)hydrazine

**DOI:** 10.1107/S1600536812042419

**Published:** 2012-10-20

**Authors:** Hoong-Kun Fun, Wan-Sin Loh, Manjunath Bhat, T. Arulmoli, G. K. Nagaraja

**Affiliations:** a X-ray Crystallography Unit, School of Physics, Universiti Sains Malaysia, 11800 USM, Penang, Malaysia; b Department of Pharmaceutical Chemistry, College of Pharmacy, King Saud University, PO Box 2457, Riyadh 11451, Saudi Arabia; c Sequent Scientific Limited, Baikampady, Karnataka, India; d Department of Chemistry, Mangalore University, Mangalagangothri, Mangalore 574 199, India

## Abstract

The title compound, C_16_H_13_Cl_2_F_3_N_2_, exists in an *E* conformation with respect to the C=N bond [1.2952 (11) Å] and the C—N—N=C torsion angle is 175.65 (8)°. The dihedral angle between the benzene rings is 42.09 (4)°. An intra­molecular C—H⋯F hydrogen bond generates an *S*(6) ring. In the crystal, the mol­ecules are linked into [101] chains by C—H⋯F hydrogen bonds.

## Related literature

For background to the properties and applications of hydrazones, see: Barbazan *et al.* (2008 ▶); Banerjee *et al.* (2009 ▶); Ghavtadze *et al.* (2008 ▶). For related structures, see: Fun *et al.* (2011*a* ▶,*b* ▶). For the stability of the temperature controller used for the data collection, see: Cosier & Glazer (1986 ▶).
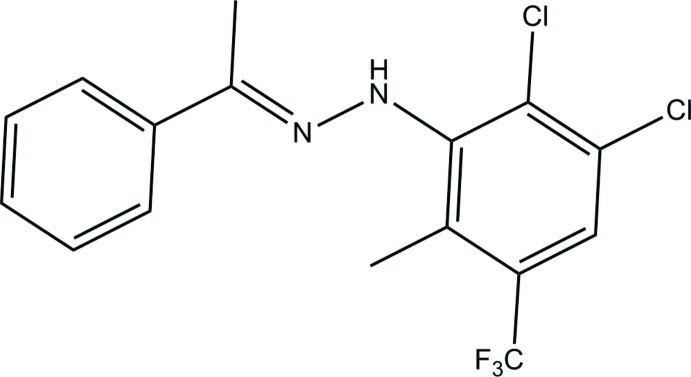


## Experimental

### 

#### Crystal data

C_16_H_13_Cl_2_F_3_N_2_
*M* *_r_* = 361.18
Monoclinic, 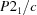

*a* = 11.2600 (16) Å
*b* = 11.4025 (17) Å
*c* = 14.8398 (16) Å
β = 123.773 (7)°
*V* = 1583.8 (4) Å^3^
*Z* = 4
Mo *K*α radiation
μ = 0.44 mm^−1^
*T* = 100 K
0.32 × 0.26 × 0.22 mm

#### Data collection

Bruker SMART APEXII Duo CCD diffractometer
Absorption correction: multi-scan (*SADABS*); Bruker, 2009 ▶) *T*_min_ = 0.872, *T*_max_ = 0.911
22269 measured reflections
5714 independent reflections
5043 reflections with *I* > 2σ(*I*)
*R*_int_ = 0.023

#### Refinement

*R*[*F* ^2^ > 2σ(*F* ^2^)] = 0.029
*wR*(*F* ^2^) = 0.090
*S* = 1.02
5714 reflections
210 parameters
H-atom parameters constrained
Δρ_max_ = 0.49 e Å^−3^
Δρ_min_ = −0.27 e Å^−3^

### 

Data collection: *APEX2* (Bruker, 2009 ▶); cell refinement: *SAINT* (Bruker, 2009 ▶); data reduction: *SAINT*; program(s) used to solve structure: *SHELXTL* (Sheldrick, 2008 ▶); program(s) used to refine structure: *SHELXTL*; molecular graphics: *SHELXTL*; software used to prepare material for publication: *SHELXTL*, *PLATON* (Spek, 2009 ▶).

## Supplementary Material

Crystal structure: contains datablock(s) global, I. DOI: 10.1107/S1600536812042419/hb6964sup1.cif

Structure factors: contains datablock(s) I. DOI: 10.1107/S1600536812042419/hb6964Isup2.hkl

Supplementary material file. DOI: 10.1107/S1600536812042419/hb6964Isup3.cml

Additional supplementary materials:  crystallographic information; 3D view; checkCIF report

## Figures and Tables

**Table 1:** Hydrogen-bond geometry (Å, °)

*D*—H⋯*A*	*D*—H	H⋯*A*	*D*⋯*A*	*D*—H⋯*A*
C1—H1*A*⋯F1^i^	0.95	2.39	3.1652 (14)	139
C15—H15*A*⋯F2	0.98	2.38	3.1018 (13)	130

Symmetry code: (i) 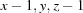.

**Fig. 1.**
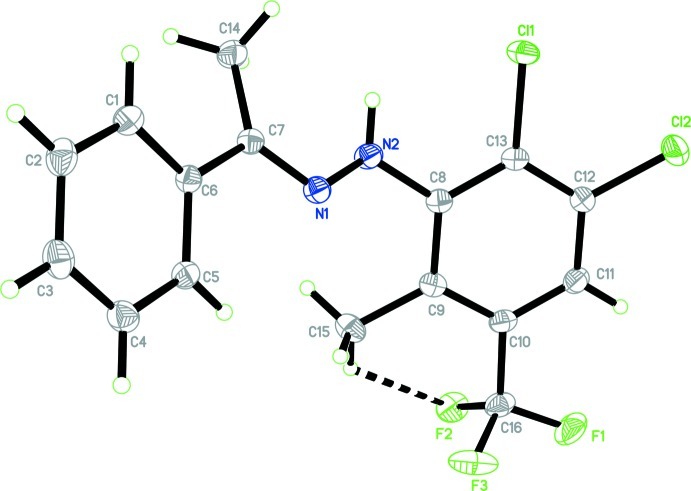
The molecular structure of the title compound, showing 50% probability displacement ellipsoids.

**Fig. 2.**
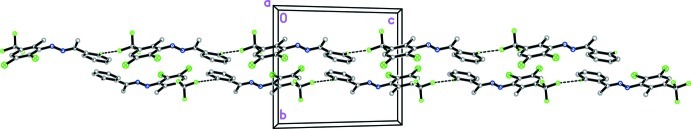
The crystal packing of the title compound, viewed along the a axis, showing the chains along [101]. H atoms not involved in the intermolecular interactions (dashed lines) have been omitted for clarity.

## supplementary crystallographic information

### Comment

Hydrazones are important compounds for drug design, as possible ligands for metal complexes, organocatalysis and also for the syntheses of heterocyclic compounds (e.g. Barbazan *et al.*, 2008; Banerjee *et al.*, 2009; Ghavtadze *et al.*, 2008). As part of our ongoing studies in this area (Fun *et al.*, 2011**a*,b*), we now describe the structure of the title compound, (I).

The title compound, as shown in Fig. 1, exists in *trans* conformation with respect to the C7═N1 bond [C7═N1 = 1.2952 (11) Å]. An *S*(6) ring is formed *via* an intramolecular C15—H15A···F2 hydrogen bond (Table 1). The dihedral angle between the benzene rings (C1–C6 & C8–C13) is 42.09 (4)°.

In the crystal, Fig. 2, the molecules are linked into chains along [101] by C1—H1A···F1 hydrogen bonds (Table 1).

### Experimental

Equimolar amount of [2,3-dichloro-6-methyl-5-(trifluoromethyl)phenyl]hydrazine and acetophenone were dissolved in a minimum amount of ethanol, then followed by the addition of 0.5 ml concentrated sulfuric acid. The solution was refluxed for 8 h then cooled to room temperature and poured into ice cold water. The solid product was collected through filtration and then dried using a drying oven at 80°C. The product was redissolved in ethanol for recrystalliziation to give yellow blocks of (I). Melting point: 368 K.

### Refinement

N-bound H atom was located from the difference Fourier map and were refined with a riding model with *U*_iso_(H) = 1.2 *U*_eq_(N) [N–H = 0.8915 Å]. The remaining H atoms were positioned geometrically and refined with a riding model with *U*_iso_(H) = 1.2 or 1.5 *U*_eq_(C) [C–H = 0.95 or 0.98 Å]. A rotating group model was applied to the methyl groups. In the final refinement, two outliners (1 2 2 and 1 2 3) were omitted.

### Crystal data

**Table:**

C_16_H_13_Cl_2_F_3_N_2_	*F*(000) = 736
*M**_r_* = 361.18	*D*_x_ = 1.515 Mg m^−^^3^
Monoclinic, *P*2_1_/*c*	Mo *K*α radiation, λ = 0.71073 Å
Hall symbol: -P 2ybc	Cell parameters from 9943 reflections
*a* = 11.2600 (16) Å	θ = 2.4–32.6°
*b* = 11.4025 (17) Å	µ = 0.44 mm^−^^1^
*c* = 14.8398 (16) Å	*T* = 100 K
β = 123.773 (7)°	Block, yellow
*V* = 1583.8 (4) Å^3^	0.32 × 0.26 × 0.22 mm
*Z* = 4	

### Data collection

**Table:**

Bruker SMART APEXII Duo CCD diffractometer	5714 independent reflections
Radiation source: fine-focus sealed tube	5043 reflections with *I* > 2σ(*I*)
Graphite monochromator	*R*_int_ = 0.023
φ and ω scans	θ_max_ = 32.6°, θ_min_ = 2.4°
Absorption correction: multi-scan (*SADABS*); Bruker, 2009)	*h* = −17→17
*T*_min_ = 0.872, *T*_max_ = 0.911	*k* = −17→16
22269 measured reflections	*l* = −22→22

### Refinement

**Table:**

Refinement on *F*^2^	Primary atom site location: structure-invariant direct methods
Least-squares matrix: full	Secondary atom site location: difference Fourier map
*R*[*F*^2^ > 2σ(*F*^2^)] = 0.029	Hydrogen site location: inferred from neighbouring sites
*wR*(*F*^2^) = 0.090	H-atom parameters constrained
*S* = 1.02	*w* = 1/[σ^2^(*F*_o_^2^) + (0.0515*P*)^2^ + 0.4114*P*] where *P* = (*F*_o_^2^ + 2*F*_c_^2^)/3
5714 reflections	(Δ/σ)_max_ = 0.001
210 parameters	Δρ_max_ = 0.49 e Å^−^^3^
0 restraints	Δρ_min_ = −0.27 e Å^−^^3^

### Special details

**Table:**

Experimental. The crystal was placed in the cold stream of an Oxford Cryosystems Cobra open-flow nitrogen cryostat (Cosier & Glazer, 1986) operating at 100.0 (1) K.
Geometry. All e.s.d.'s (except the e.s.d. in the dihedral angle between two l.s. planes) are estimated using the full covariance matrix. The cell e.s.d.'s are taken into account individually in the estimation of e.s.d.'s in distances, angles and torsion angles; correlations between e.s.d.'s in cell parameters are only used when they are defined by crystal symmetry. An approximate (isotropic) treatment of cell e.s.d.'s is used for estimating e.s.d.'s involving l.s. planes.
Refinement. Refinement of *F*^2^ against ALL reflections. The weighted *R*-factor *wR* and goodness of fit *S* are based on *F*^2^, conventional *R*-factors *R* are based on *F*, with *F* set to zero for negative *F*^2^. The threshold expression of *F*^2^ > σ(*F*^2^) is used only for calculating *R*-factors(gt) *etc*. and is not relevant to the choice of reflections for refinement. *R*-factors based on *F*^2^ are statistically about twice as large as those based on *F*, and *R*- factors based on ALL data will be even larger.

### Fractional atomic coordinates and isotropic or equivalent isotropic displacement parameters (Å^2^)

**Table:**

	*x*	*y*	*z*	*U*_iso_*/*U*_eq_	
Cl1	0.07133 (2)	0.57463 (2)	0.919947 (18)	0.02243 (6)	
Cl2	0.27412 (3)	0.51560 (3)	1.16881 (2)	0.03137 (7)	
F1	0.76376 (7)	0.64100 (7)	1.26985 (6)	0.04073 (19)	
F2	0.72355 (7)	0.79611 (5)	1.17471 (5)	0.02773 (13)	
F3	0.76971 (7)	0.63239 (6)	1.12760 (7)	0.03906 (18)	
N1	0.24945 (8)	0.64196 (7)	0.75151 (6)	0.01758 (13)	
N2	0.21577 (8)	0.68019 (7)	0.82355 (6)	0.01970 (14)	
H1	0.1231	0.6830	0.7982	0.024*	
C1	0.08557 (10)	0.60301 (8)	0.46427 (7)	0.02097 (16)	
H1A	−0.0116	0.6165	0.4387	0.025*	
C2	0.11987 (11)	0.56352 (9)	0.39242 (8)	0.02343 (17)	
H2A	0.0464	0.5506	0.3184	0.028*	
C3	0.26177 (11)	0.54303 (8)	0.42924 (8)	0.02305 (17)	
H3A	0.2855	0.5160	0.3806	0.028*	
C4	0.36909 (10)	0.56246 (8)	0.53808 (8)	0.02125 (16)	
H4A	0.4660	0.5484	0.5634	0.026*	
C5	0.33508 (9)	0.60236 (7)	0.60973 (7)	0.01782 (15)	
H5A	0.4090	0.6157	0.6836	0.021*	
C6	0.19245 (9)	0.62302 (7)	0.57366 (7)	0.01647 (14)	
C7	0.15569 (9)	0.66423 (7)	0.65008 (7)	0.01714 (14)	
C8	0.30910 (9)	0.65100 (7)	0.93301 (7)	0.01651 (14)	
C9	0.45661 (9)	0.67664 (7)	0.98943 (7)	0.01764 (15)	
C10	0.54176 (9)	0.65007 (7)	1.10045 (7)	0.01872 (15)	
C11	0.48613 (10)	0.60149 (8)	1.15569 (7)	0.02078 (16)	
H11A	0.5468	0.5845	1.2309	0.025*	
C12	0.34106 (10)	0.57840 (8)	1.09936 (7)	0.01957 (15)	
C13	0.25253 (9)	0.60334 (8)	0.98898 (7)	0.01731 (14)	
C14	0.01767 (10)	0.72840 (9)	0.60945 (8)	0.02298 (17)	
H14A	0.0361	0.7958	0.6565	0.034*	
H14B	−0.0503	0.6753	0.6102	0.034*	
H14C	−0.0225	0.7558	0.5353	0.034*	
C15	0.51547 (11)	0.73642 (9)	0.93086 (8)	0.02373 (17)	
H15A	0.6001	0.7824	0.9828	0.036*	
H15B	0.4426	0.7885	0.8747	0.036*	
H15C	0.5418	0.6770	0.8972	0.036*	
C16	0.69874 (10)	0.67904 (8)	1.16736 (8)	0.02398 (18)	

### Atomic displacement parameters (Å^2^)

**Table:**

	*U*^11^	*U*^22^	*U*^33^	*U*^12^	*U*^13^	*U*^23^
Cl1	0.01517 (9)	0.02920 (11)	0.02257 (11)	−0.00304 (7)	0.01028 (8)	−0.00384 (7)
Cl2	0.02648 (12)	0.04378 (15)	0.02537 (12)	−0.00394 (10)	0.01537 (10)	0.00792 (10)
F1	0.0198 (3)	0.0430 (4)	0.0341 (4)	−0.0034 (3)	−0.0007 (3)	0.0151 (3)
F2	0.0243 (3)	0.0190 (3)	0.0311 (3)	−0.0060 (2)	0.0099 (2)	−0.0040 (2)
F3	0.0199 (3)	0.0342 (3)	0.0623 (5)	−0.0027 (2)	0.0223 (3)	−0.0143 (3)
N1	0.0180 (3)	0.0189 (3)	0.0170 (3)	0.0008 (2)	0.0105 (3)	−0.0012 (2)
N2	0.0173 (3)	0.0259 (4)	0.0161 (3)	0.0032 (3)	0.0094 (3)	−0.0008 (3)
C1	0.0184 (4)	0.0250 (4)	0.0172 (4)	−0.0010 (3)	0.0084 (3)	−0.0001 (3)
C2	0.0270 (4)	0.0244 (4)	0.0171 (4)	−0.0028 (3)	0.0111 (3)	−0.0015 (3)
C3	0.0314 (5)	0.0200 (4)	0.0232 (4)	0.0004 (3)	0.0185 (4)	−0.0007 (3)
C4	0.0228 (4)	0.0206 (4)	0.0244 (4)	0.0031 (3)	0.0157 (4)	0.0015 (3)
C5	0.0176 (3)	0.0176 (3)	0.0181 (3)	0.0016 (3)	0.0099 (3)	0.0014 (3)
C6	0.0168 (3)	0.0161 (3)	0.0162 (3)	0.0004 (3)	0.0090 (3)	0.0011 (3)
C7	0.0163 (3)	0.0177 (3)	0.0179 (3)	0.0009 (3)	0.0098 (3)	0.0005 (3)
C8	0.0161 (3)	0.0164 (3)	0.0171 (3)	0.0004 (3)	0.0092 (3)	−0.0019 (3)
C9	0.0168 (3)	0.0158 (3)	0.0209 (4)	−0.0007 (3)	0.0109 (3)	−0.0021 (3)
C10	0.0144 (3)	0.0156 (3)	0.0222 (4)	0.0002 (3)	0.0077 (3)	−0.0005 (3)
C11	0.0181 (4)	0.0196 (4)	0.0195 (4)	0.0005 (3)	0.0072 (3)	0.0025 (3)
C12	0.0185 (4)	0.0203 (4)	0.0199 (4)	−0.0006 (3)	0.0106 (3)	0.0016 (3)
C13	0.0144 (3)	0.0185 (3)	0.0183 (4)	−0.0005 (3)	0.0086 (3)	−0.0019 (3)
C14	0.0196 (4)	0.0267 (4)	0.0234 (4)	0.0066 (3)	0.0125 (3)	0.0042 (3)
C15	0.0228 (4)	0.0257 (4)	0.0258 (4)	−0.0059 (3)	0.0155 (4)	−0.0041 (3)
C16	0.0165 (4)	0.0191 (4)	0.0288 (4)	−0.0009 (3)	0.0079 (3)	0.0006 (3)

### Geometric parameters (Å, º)

**Table:**

Cl1—C13	1.7309 (9)	C5—H5A	0.9500
Cl2—C12	1.7340 (10)	C6—C7	1.4833 (12)
F1—C16	1.3414 (12)	C7—C14	1.5075 (12)
F2—C16	1.3558 (11)	C8—C13	1.4069 (12)
F3—C16	1.3394 (13)	C8—C9	1.4139 (12)
N1—C7	1.2952 (11)	C9—C10	1.4032 (13)
N1—N2	1.3884 (10)	C9—C15	1.5165 (13)
N2—C8	1.3985 (11)	C10—C11	1.3936 (13)
N2—H1	0.8915	C10—C16	1.5060 (13)
C1—C2	1.3951 (13)	C11—C12	1.3850 (13)
C1—C6	1.4013 (12)	C11—H11A	0.9500
C1—H1A	0.9500	C12—C13	1.3936 (12)
C2—C3	1.3912 (15)	C14—H14A	0.9800
C2—H2A	0.9500	C14—H14B	0.9800
C3—C4	1.3958 (14)	C14—H14C	0.9800
C3—H3A	0.9500	C15—H15A	0.9800
C4—C5	1.3916 (12)	C15—H15B	0.9800
C4—H4A	0.9500	C15—H15C	0.9800
C5—C6	1.4020 (12)		
			
C7—N1—N2	115.99 (7)	C11—C10—C9	122.64 (8)
N1—N2—C8	117.97 (7)	C11—C10—C16	116.48 (8)
N1—N2—H1	116.3	C9—C10—C16	120.81 (8)
C8—N2—H1	116.5	C12—C11—C10	119.05 (8)
C2—C1—C6	120.91 (8)	C12—C11—H11A	120.5
C2—C1—H1A	119.5	C10—C11—H11A	120.5
C6—C1—H1A	119.5	C11—C12—C13	120.24 (8)
C3—C2—C1	119.96 (9)	C11—C12—Cl2	118.45 (7)
C3—C2—H2A	120.0	C13—C12—Cl2	121.31 (7)
C1—C2—H2A	120.0	C12—C13—C8	120.66 (8)
C2—C3—C4	119.63 (9)	C12—C13—Cl1	119.65 (7)
C2—C3—H3A	120.2	C8—C13—Cl1	119.69 (7)
C4—C3—H3A	120.2	C7—C14—H14A	109.5
C5—C4—C3	120.47 (9)	C7—C14—H14B	109.5
C5—C4—H4A	119.8	H14A—C14—H14B	109.5
C3—C4—H4A	119.8	C7—C14—H14C	109.5
C4—C5—C6	120.44 (8)	H14A—C14—H14C	109.5
C4—C5—H5A	119.8	H14B—C14—H14C	109.5
C6—C5—H5A	119.8	C9—C15—H15A	109.5
C1—C6—C5	118.60 (8)	C9—C15—H15B	109.5
C1—C6—C7	120.82 (8)	H15A—C15—H15B	109.5
C5—C6—C7	120.57 (7)	C9—C15—H15C	109.5
N1—C7—C6	115.64 (7)	H15A—C15—H15C	109.5
N1—C7—C14	123.57 (8)	H15B—C15—H15C	109.5
C6—C7—C14	120.78 (7)	F3—C16—F1	106.72 (9)
N2—C8—C13	118.89 (8)	F3—C16—F2	106.25 (8)
N2—C8—C9	121.03 (8)	F1—C16—F2	105.61 (8)
C13—C8—C9	119.91 (8)	F3—C16—C10	113.04 (8)
C10—C9—C8	117.49 (8)	F1—C16—C10	112.18 (8)
C10—C9—C15	122.61 (8)	F2—C16—C10	112.51 (8)
C8—C9—C15	119.82 (8)		
			
C7—N1—N2—C8	175.65 (8)	C15—C9—C10—C11	176.09 (8)
C6—C1—C2—C3	0.26 (14)	C8—C9—C10—C16	−177.55 (8)
C1—C2—C3—C4	−0.15 (14)	C15—C9—C10—C16	−0.72 (13)
C2—C3—C4—C5	−0.14 (14)	C9—C10—C11—C12	−0.09 (14)
C3—C4—C5—C6	0.32 (13)	C16—C10—C11—C12	176.85 (8)
C2—C1—C6—C5	−0.09 (13)	C10—C11—C12—C13	0.13 (14)
C2—C1—C6—C7	−179.58 (8)	C10—C11—C12—Cl2	178.90 (7)
C4—C5—C6—C1	−0.21 (13)	C11—C12—C13—C8	0.68 (13)
C4—C5—C6—C7	179.29 (8)	Cl2—C12—C13—C8	−178.05 (7)
N2—N1—C7—C6	−179.96 (7)	C11—C12—C13—Cl1	−179.68 (7)
N2—N1—C7—C14	0.51 (13)	Cl2—C12—C13—Cl1	1.59 (11)
C1—C6—C7—N1	157.04 (8)	N2—C8—C13—C12	−176.86 (8)
C5—C6—C7—N1	−22.44 (12)	C9—C8—C13—C12	−1.53 (13)
C1—C6—C7—C14	−23.41 (12)	N2—C8—C13—Cl1	3.50 (11)
C5—C6—C7—C14	157.11 (8)	C9—C8—C13—Cl1	178.83 (6)
N1—N2—C8—C13	−131.65 (8)	C11—C10—C16—F3	127.38 (9)
N1—N2—C8—C9	53.07 (11)	C9—C10—C16—F3	−55.61 (12)
N2—C8—C9—C10	176.75 (8)	C11—C10—C16—F1	6.65 (12)
C13—C8—C9—C10	1.52 (12)	C9—C10—C16—F1	−176.35 (8)
N2—C8—C9—C15	−0.17 (12)	C11—C10—C16—F2	−112.26 (10)
C13—C8—C9—C15	−175.40 (8)	C9—C10—C16—F2	64.74 (12)
C8—C9—C10—C11	−0.74 (13)		

### Hydrogen-bond geometry (Å, º)

**Table:**

*D*—H···*A*	*D*—H	H···*A*	*D*···*A*	*D*—H···*A*
C1—H1*A*···F1^i^	0.95	2.39	3.1652 (14)	139
C15—H15*A*···F2	0.98	2.38	3.1018 (13)	130

Symmetry code: (i) *x*−1, *y*, *z*−1.
